# The Genuine Works of Hippocrates

**Published:** 1886-10

**Authors:** 


**The Genuine Works of Hippocrates.** Translated from the Greek, with a Preliminary Discourse and Annotations. By FRANCIS ADAMS, LL. D., Surgeon. Volume II. Being Vol. VII. of Wood's Library for 1886. New York: William Wood & Co.

In noticing the first volume we expressed the opinion that this admirable translation would be a valuable addition to Wood's Library, and this second volume fully confirms our opinion. It will be read with interest by many.

